# Linking the epidemiology of coccidioidomycosis and environmental exposure through targeted genomic enrichment of *Coccidioides posadasii*

**DOI:** 10.1128/mbio.03396-25

**Published:** 2025-12-30

**Authors:** Jason W. Sahl, Nathan E. Stone, Daniel R. Kollath, Marieke Ramsey, Rebecca Ballard, Ana Braga, Amber I. Jones, Pierre Herckes, Matthew P. Fraser, Amelia Stout, Bridget M. Barker, Paul Keim, David M. Wagner

**Affiliations:** 1 Pathogen and Microbiome Institute, Northern Arizona University 3356, Flagstaff, Arizona, USA; 2 School of Sustainable Engineering and the Built Environment, Arizona State University 7864, Tempe, Arizona, USA; Instituto Carlos Chagas, Curitiba, Brazil

**Keywords:** *Coccidioides*, DNA enrichment, Valley fever

## Abstract

Valley fever, a disease caused by *Coccidioides* spp., is a fungal respiratory disease with an expanding range. Methods to culture the pathogen from soil, especially *Coccidioides posadasii*, are very challenging, limiting the genomics knowledge of environmental strains. In this study, we designed and tested a targeted DNA capture and enrichment system for the characterization of *Coccidioides* genomes without the need to culture. In this system, RNA probes are hybridized to *Coccidioides* DNA in a complex sample, followed by DNA amplification, sequencing, and analysis. Our enrichment system was targeted toward coding region sequences in *C. posadasii* str. Silveira and tested on control DNA spiked into soil; DNA hybridized to probes was then sequenced and correctly placed into a reference phylogeny, based on the known placement of the whole-genome sequence. We then applied the enrichment system to a range of sample types (soil, air filters, rodent tissue) from a site in Mesa, Arizona, USA. The enriched samples were sequenced and placed into the *C. posadasii* phylogeny to understand the phylogenetic diversity within the Mesa site over time. The results demonstrate that low DNA signal in most sample types was boosted after enrichment. Enriched sequences from air filters collected at multiple time points from the Mesa site linked two different isolates collected from fatal cases of Coccidioidomycosis in a pig-tailed macaque colony housed at the Mesa site. This represents the first time that environmental *C. posadasii* DNA was directly linked to Coccidioidomycosis and demonstrates the power of this approach for genomic epidemiology.

**IMPORTANCE:**

All human cases of Valley fever are acquired through environmental exposure, so surveillance and characterization of the pathogen in soil are critical for risk mitigation efforts. Current databases are biased toward human clinical isolates, and little is known about the genomics of environmental strains of *Coccidioides posadasii*. In this study, we designed, tested, and validated a probe enrichment system that amplifies trace DNA in a complex sample. Sequenced DNA can be used to link environmental exposure with human cases, directing public health agencies to interventions that limit human exposure. This use case was demonstrated in this study, as trace DNA trapped on air filters was linked to a fatal case of primate Coccidioidomycosis at a site in Arizona. The probe enrichment system described in this study represents a powerful tool to better understand the genomic composition of environmental *C. posadasii* strains, which can aid in public health investigations.

## INTRODUCTION

Valley fever is a respiratory disease caused by two species within *Coccidioides, C. posadasii* and *C. immitis* (1). *C. posadasii* is largely found in Arizona and central/south America, although the range is thought to be expanding due to climate change (2). *Coccidioides* spp. have a dimorphic life cycle, with both saprobic and parasitic phases (1). When a mammal inhales the arthroconidia, most likely from blowing dust, the pathogen forms spherules that contain multiple endospores. When the spherule ruptures, the endospores are released, continuing the parasitic life cycle, and—when the animal dies—potentially creating an environmental reservoir for future exposure. Thus, animals, through infection, movement, and death, are likely key to the persistence and dissemination of the pathogen (3, 4). Based on its lifecycle, almost all cases of Coccidioidomycosis are acquired from environmental exposure and not human-to-human transmission.

The preferred habitat for *Coccidioides* appears to be hot, dry soils, including those near animal burrows (5, 6). *Coccidioides* has been found in relatively shallow soils (2–20 cm) (7), suggesting that wind could carry the pathogen, thereby exposing both humans and non-human animals via the respiratory route. Although at least one study has suggested a lack of correlation between dust storms and Valley fever (8), others have reported an association between wind gusts, temperature, and soil moisture with the environmental presence of *Coccidioides* (9). Previous air sampling in Phoenix, Arizona, demonstrated an uneven and seasonal spatial distribution of *Coccidioides* in the environment (10).

Most studies of *Coccidioides* detection in the environment have focused on molecular assays detecting the presence of *Coccidioides* DNA in extracts obtained from complex backgrounds (e.g., soil) using ITS2 (11), or more recent assays targeting repetitive sequences including Cocci-Dx (12) and Cocci-Env (13). Genotyping from the environment is typically limited to the information within single genes, which fails to provide meaningful resolution between diverse strains (14). Although *C. immitis* has been successfully cultured from soil (15), isolation of environmental *C. posadasii* requires passage through mice, and even this is not always successful (16). As such, almost all *C. posadasii* genome sequencing efforts to date have been limited to clinical isolates. These limitations have resulted in a lack of genomics data from environmental isolates that severely limits our understanding of the genomic composition of environmental strains. For clinical cases, a lack of environmental genomes complicates source attribution efforts that would focus on risk mitigation efforts.

Our research group has used probe enrichment systems to amplify and analyze DNA in complex samples from a range of pathogens, including *Yersinia pestis* (17, 18), *Francisella spp*. (19), and *Leptospira* spp. (20). Probe enrichment/capture systems allow for the amplification of low-abundance and degraded target DNA that can then be used to address important biological questions. Analyses that can be addressed using enrichment data that cannot be addressed by marker gene data include high-resolution genotyping, outbreak analysis and contact tracing, analysis of insertions/deletions that could have a biological impact, and gene content/differences.

The goal of this study was to develop, test, and validate a probe capture enrichment system for genotyping *Coccidioides* from complex environmental samples. The probe enrichment system allows for high-resolution genotyping, revealing genomic information for previously unknown environmental strains of *C. posadasii*. In this study, we analyzed multiple sample types from a single site in Mesa, Arizona, USA, and identified links between enriched air filter samples and animal tissues from fatal cases of Coccidioidomycosis. These results demonstrate the utility of our probe enrichment system on environmental samples in linking disease events in humans and other susceptible hosts to environmental sources.

## MATERIALS AND METHODS

### Reference genome set

*C. posadasii* reference genome assemblies (*n* = 13) were downloaded with the ncbi-genome-download tool (https://github.com/kblin/ncbi-genome-download) on January 25th, 2024. We also downloaded 220 SRA data sets and assembled them with Spades v3.15.5 (21); of these, 168 generated assemblies of the correct size for downstream analyses. Details for all external genomes are shown in Table S1. We also downloaded all *C. immitis* genome assemblies (*n* = 5) with the ncbi-genome-download tool. We then downloaded 82 *C*. *immitis* SRA data sets, assembled them with SPAdes, and discarded four for poor quality, resulting in a set of 78 SRA genome assemblies (Table S1).

### RNA probe design and validation

The *C. posadasii* Silveira genome assembly (GCA_018416015.2) (22) was sliced into 120 nt fragments overlapping by 60 nts. Candidate probes were aligned against public *C. posadasii* genome assemblies (Table S1) with BLAT v35 x 1 (23) in conjunction with LS-BSR v1.3 (24). Probes were kept that had a blast score ratio (BSR) (25) value ≥0.8 in all reference genomes (core genome probes), resulting in a set of 603,202 candidates. The core genome probes were then aligned against Silveira CDSs (*n* = 8,516; 12,149,995 nts) with minimap2 v2.29 (26) and only aligned probes were kept, resulting in a set of 300,055 candidates. Remaining probes were aligned against a set of near neighbor genomes, including *Uncinocarpus reesii* 1704 (GCA_000003515.2), *Paracoccidioides brasilensis* (GCA_001713695.1, GCA_001713645.1), *Blastomyces gilchristii* (GCF_000003855.2), and a set of nine *Eurotiales* sp. genomes from Arizona (B. M. Barker, unpublished data)*;* any probe that had a BSR value >0.7 was removed from the probe set for potential cross-reactivity. The final design consisted of 292,408 probes (27). To calculate the percentage of the Silveira genome covered, probes were aligned to the Silveira assembly with minimap2, and the breadth of coverage (BOC) was calculated with Samtools v1.6 (28) at a minimum depth of 1×.

### Reference population structure of *C. posadasii*

To determine the *C. posadasii* population structure, simulated reads were generated from reference genome assemblies (*n* = 190) with art-illumina v2.5.8 (29) and aligned against the Silveira assembly with minimap2; only assemblies were used to normalize the variable sample types. Single-nucleotide polymorphisms (SNPs) were called from the BAM files with GATK v4.5.0.0 (30) using the following settings “-ploidy 1 --include-non-variant-sites.” SNPs that fell within duplicated regions, based on a reference self-alignment with NUCmer v3.1 (31), were filtered from downstream analyses. SNPs were then collated into a matrix with NASP v1.2.0 (32). For rooting, SNPs were queried in *C. immitis* WA-211 (GCA_004115165.2) with a custom script (https://gist.github.com/jasonsahl/d1472d34198e857ed6594d3505cf55b9). A phylogeny was inferred on the concatenated set of parsimony-informative SNPs (*n* = 287,010) with IQ-TREE v 2.3.6 (33) in conjunction with the integrated ModelFinder (34). The resulting phylogeny was visualized in Figtree v1.4.4 (http://tree.bio.ed.ac.uk/software/figtree/).

### Samples enriched

To test our DNA capture and enrichment system on complex environmental samples, we used DNA extracts from soil and rodent tissue samples that were qPCR positive for *Coccidioides* spp. DNA. Sample 407-B16bi-R3 is a soil sample collected near Tucson, Arizona, in May of 2018 (16). Sample 14-2A is a soil sample that was collected near the Washington National Primate Research Center (WaNPRC) in Mesa, Arizona, in July of 2022 and tested positive for *Coccidioides* by Cocci-Dx. Sample 31L was obtained from *Peromyscus eremicus* (cactus mouse) lung tissue; the mouse was collected on the WaNPRC as a result of pest management efforts and was collected under the State of Arizona scientific collecting permit #SP627992. In an effort to validate our enrichment method in animal tissues, laboratory mice were experimentally infected with *C. posadasii* strains Silveria and CPA00032; *C. posadasii* strain Silveira is a common lab strain that was collected from a patient in 1951 (35), and CPA00032 is a mouse-passaged soil isolate collected from Tucson, Arizona (16).

For the air filter collection, two Tisch Environmental PM10 high-volume air samplers were deployed to collect 24-hour time-integrated samples at the WaNPRC. The samplers collected particulate matter with an aerodynamic diameter less than or equal to 10 µm. One of the samplers was equipped with pre-fired (550°C overnight) quartz fiber filters (QFFs), and the other sampler with cellulose filters. Phenol-chloroform DNA extractions were performed on QFFs (details provided below).

For the laboratory mouse infections, arthroconidia were harvested from 8-week-old *C. posadasii* strains Silveira and CPA00032 using methods described in reference (36). Concentrated arthroconidia stocks were diluted to an inoculum of 500 colony-forming units (CFU) per 30 µL. 8-week-old male C57/B6 mice were anesthetized using ketamine/xylazine (80/8 mg/kg) and intranasally infected with 500 CFU of each fungal strain in 30 µL. Mice were euthanized when considered moribund, defined by weight loss of ≥20% from initial body weight, or a combination of signs such as inactivity, ataxia, ruffled fur, and dehydration. Following euthanasia, the lungs were harvested for DNA extraction.

### DNA capture and enrichment

Prior to DNA capture and enrichment, all DNA extracts were assessed for quality and quantity by Qubit BR or HS dsDNA kits (Thermo Fisher Scientific, Waltham, MA, USA) and Fragment Analyzer genomic DNA analysis kits (Agilent Technologies, Santa Clara, CA, USA). DNAs were then diluted to ≤4 ng/µL in 30–50 µL, sheared using a Q800 Sonicator (QSonica, Newtown, CT) at a protocol of 60% amplitude, 15 s on/off to obtain an optimal fragment size (150–450 bp) for the capture step, and then unique dual-indexed libraries were prepared for each sample according to the SureSelect XT-low input Target Enrichment System protocol (Agilent Technologies, Santa Clara, CA, USA). Briefly, end repair and A-tailing were performed on sheared DNA, and an adaptor with a barcode was ligated to the A-tailed ends of the DNA fragments, followed by purification. Each ligated fragment was uniquely dual-indexed via PCR amplification for 14 cycles (2 minutes at 98°C, 14 cycles for 30 s at 98°C, 30 s at 60°C, 1 minutes at 72°C, and a final extension of 5 minutes at 72°C), followed by purification. All DNA purification steps were carried out using Agencourt AMPure XP beads (0.8–1× bead ratio; Beckman Coulter Genomics, Brea, CA). Library quantity was assessed by Qubit Br dsDNA, and the size and quality were assessed by Fragment Analyzer. To achieve a final yield of 1000–2000 ng per sample, libraries were re-amplified in four replicates for 8–14 cycles, and all replicates were pooled. A slow hybridization method was implemented to prevent dissociation of probes from AT-rich regions, with ~1,000–2,000 ng of each library hybridized at 67.5°C for 16–24 hours. Libraries were then subjected to one, two, or three rounds of enrichment; for round two and three enrichments, ~1,000 ng of input DNA was used. Hybridized libraries were recovered using 50 µL of Dynabeads from the MyOne Streptavidin T1 Kit (Thermo Fisher Scientific, Waltham, MA) according to the SureSelect protocol. We then PCR-amplified directly from the beads using the SureSelectXT-LI Primer Mix, using the same PCR conditions described above for library preparation except with 14 cycles. The amplicons were then separated from the beads via a magnetic plate and transferred to a new tube. A second PCR was conducted from the beads using a KAPA HiFi PCR ready mix (Roche KAPA Biosystems, Wilmington) to amplify the residual capture library, combined with the first PCR event, and purified.

We confirmed the presence or absence of *Coccidioides* DNA in each sample library prior to enrichment using the Cocci-Dx assay (12), and also assessed the increase in the proportion of *Coccidioides* DNA after each round of enrichment using a novel qPCR assay (CocciProbe) designed to target a conserved probe in the capture and enrichment system. This assay utilizes primer pair CocciProbe_34F (5′-CCATTGCAGTAAGCAGTGGT-3′) and CocciProbe_110R (5′-GTCGAAGATTCGAGTGTGAGC-3′); this sequence was derived from a gene (CPSG_0526; cimg_00509) that is thought to be unique to *Coccidioides* (37). The CocciProbe assay was performed on the sequence-ready libraries prior to enrichment using ~20 ng of input DNA, and also after each round of enrichment using ~1 ng of input DNA. When the qPCR cycle threshold (Ct) for enriched libraries was equivalent to the ~1 ng dilution of our Silveira gDNA control, sample enrichment was presumed to be complete. PCRs were carried out in 10 µL volumes containing the following reagents (given in final concentrations): 1 µL of diluted DNA template, 1× SYBR Green Universal master mix (Applied Biosystems, Foster City, CA, USA), and 0.4 µM of each primer. The assay was run on an Applied Biosystems 7500 Fast Real-Time PCR System with SDS 7500 software v2.0.6 under the following conditions: 50°C for 2 minutes, 95°C for 10 minutes, and 40 cycles of 95°C for 15 s and 58°C for 1 minute; positive and non-template controls were included on all runs.

### Pig-tailed macaque isolates

Tissues (lungs, spleens, trachea, liver, and lymph nodes) from naturally infected pig-tailed macaques (PTM; *Macaca nemestrina*) from the WaNPRC were harvested from euthanized animals that succumbed to Coccidioidomycosis to confirm infection and complete diagnostics; macaques were housed at the WaNPRC in Mesa, Arizona, USA, and naturally acquired the infection from the environment. Subsections of tissues were placed in a bead tube with 1.5 mm zirconium beads (BeadBug, Millipore Sigma) in 1 mL of 1× phosphate-buffered saline and homogenized at 5 m/s for 1 minute. 50 µL of homogenate was placed onto 2× glucose yeast extract (GYE) media supplemented with 1 mg/mL of penicillin-streptomycin antibiotics and incubated at 37°C. Fungal colonies were passaged (×3) onto clean media plates until pure.

### DNA extraction and sequencing

DNA was extracted from the soil samples using the DNeasy PowerSoil Pro kit (QIAGEN, Valencia, CA). Due to the potential infectious propagules in the soil, DNA extractions were performed in a biological safety level two laboratory inside a biological safety cabinet. The manufacturer’s protocol was followed, except an additional 10-minute heat step at 65°C was added before the homogenization step. Two DNA extractions were performed for each sample. Approximately 250 mg of soil was placed into each tube with lysis buffer. Purified DNA was stored at −20°C.

DNA was extracted from rodent lung tissue, air filters, as well as purified isolates using a phenol-based extraction. The whole lung was placed in a tube with 1.5 mm zirconium beads (BeadBug, Millipore Sigma) in 1 mL of fungal-specific lysis buffer consisting of molecular-grade water, 3M NaOAc at pH 6.0, 10% SDS, 0.5 M EDTA at pH 8.0, and 1M Tris HCl at pH 7.5. The tissues were incubated at 37°C for 30 minutes. 500 µL of phenol:chloroform isoamyl (1:1) was added to the bead tube and homogenized for 2 minutes (30 s on, 30 s rest) at 6 m/s using a Fisherbrand Bead Mill 24 homogenizer (Thermo Fisher Scientific). Samples were centrifuged at 13,000 × *g* for 4 minutes. The aqueous layer was removed and washed with chloroform isoamyl (1:1) and centrifuged at 13,000 × *g* for 4 minutes. The aqueous layer was again removed, and DNA was precipitated with 600 µL of cold isopropanol and 60 µL 3M Na-Acetate and stored at −20°C for 30 minutes. Samples were centrifuged at 4°C at 13,000 × *g* for 20 minutes; the isopropanol was then decanted, and cold 100% ethanol was added. Samples were centrifuged at 4°C at 13,000 × *g* for 5 minutes, the ethanol was decanted, and the DNA pellet was allowed to dry for 20 minutes. The dry DNA pellet was resuspended in molecular-grade water.

DNA library construction for enrichment and whole-genome sequencing was performed using the KAPA Hyper Prep Kits for Illumina NGS platforms and KAPA UDI Primer Mixes per manufacturer’s protocol, with double-sided size selection performed after sonication. Final libraries for enrichments and whole-genome sequences were quantified on an Applied Biosystems QuantStudio 7 Flex Real-Time PCR System using the KAPA SYBR FAST ROX Low qPCR Master Mix for Illumina platforms. The libraries were pooled together at equimolar concentrations, and quality was assessed with Agilent Technologies TapeStation 4150 System. Final quantification by qPCR preceded sequencing of the final library. Samples were sequenced on the Illumina NextSeq1000 using the NextSeq1000/2000 P1 XLEAP-SBS Reagent Kit using the standard Illumina protocol.

### BOC analysis

The BOC, or the amount of a reference sequence covered by a minimum number of reads, was calculated by aligning raw sequence reads against reference genomes/genes with minimap2 and calculating coverage with SAMtools at a minimum depth of 5×.

### *WG-FAST* analysis

The whole-genome focused array SNP typing (*WG-FAST*) method can be used to genotype from partial SNP data sets (38) by querying SNPs from sequence data, and then placing the sample into an existing phylogenetic framework with the evolutionary placement algorithm (39) incorporated into RAxML v8.12 (40). Samples were processed in this study using *WG-FAST* v1.3 and the following commands: “-s F -j GTRGAMMA -c 2 -p 4 -o 0.8.” The reference files used for each *WG-FAST* analysis are publicly available (https://github.com/jasonsahl/cocci_wgfast); these files include the FASTA file for Silveira, the NASP SNP matrix, and the IQ-TREE inferred on the concatenation of parsimony-informative SNPs. The consistency and retention index values (41), which demonstrate how consistent the SNP data are with the tree, were calculated with Phangorn v2.11.1 (42).

### Identification of *C. posadasii*specific probes

All probes were aligned against Silveira and *C. immitis* str. RS with LS-BSR in conjunction with BLAT. Probes were identified that had a BSR value ≥0.8 in *C. posadasii* and <0.4 in *C. immitis*. Probes that were specific to Silveira were then aligned against a larger set of *C. immitis* genomes to confirm a lack of alignment (BSR value <0.4).

### *In silico* limit of detection for *WG-FAST* placement

Reads were simulated with art-illumina from the assembly of HS-I-000233 (SRR14250212), resulting in 8.24M reads. Reads were aligned against probes with minimap2, and only reads that aligned were then extracted with SAMtools, resulting in a set of 4.06M reads. From this sample set, reads were sampled at different levels (100–1,000, step by 100) using Seqtk v1.3 (https://github.com/lh3/seqtk). Subsampled reads were inserted into the *WG-FAST* phylogeny, and the correct number of placements, based on a visual inspection of the phylogeny, was calculated.

### Gene content

As a proof of concept, mutations were identified by aligning reads from 407-B16bi-R3 (SRR30946007) against Silveira with Snippy v4.6.0 (https://github.com/tseemann/snippy). In addition, reads that mapped against Silveira were assembled with SPAdes, and the genome was annotated by BRAKER3 v3.0.8 (43).

### *In vitro* assessment of probe enrichment for *C. immitis*

To generate reference files for *WG-FAST*, 82 *C*. *immitis* genome assemblies (Table S1) were aligned against *C. immitis* WA-211 (GCA_004115165.2) with NASP. A maximum-likelihood phylogeny was inferred on the concatenated SNP alignment with IQ-TREE.

Reads from *C. immitis* B14131 (SRR21204705) were mapped against enrichment probes, and only mapped reads were exported from the binary alignment map (BAM) file (*n* = 6.5M paired reads). Mapped and sub-sampled reads were then aligned against WA-211 and inserted into the *C. immitis* phylogeny with *WG-FAST*.

## RESULTS

### Composition of the probe enrichment system

The final probe design consisted of 292,408 probes. Mapping probes against the Silveira genome demonstrated that 39.54% (11,147,083 bases) of the total genome, and 82% of the sequence within CDSs (9,961,001 bases) were covered by the probe set at a depth of 1×. Although obtaining the entire genome may be useful for some research questions, the upper limit on the number of RNA probes that can be synthesized in Agilent’s largest Tier 5 design restricted our analysis to the CDSs. The flexible nature of the enrichment system would allow for shifting the probes to different regions of the genome or doubling the enrichment probes (two Tier 5 designs) and merging to cover most of the target genome.

### *C. posadasii*-specific probes

A total of 483 probes within 61 CDSs (Table S2) were identified that mapped uniquely to *C. posadasii* (27). Sequence reads from most samples enriched in this study mapped against the *C. posadasii*-specific probes (Table S3), although the probe design process, combined with probe hybridization, suggests that the system should also work for *C. immitis;* mapping probes against the *C. immitis* WA-211 (GCA_004115165.2) genome demonstrated that 40.2% of the genome (1× depth) was expected to be covered by the probe set, and a *WG-FAST* analysis demonstrated a close link of a subsampled data set (Fig. S1).

### *C. posadasii* population structure

From a reference set of 191 *C*. *posadasii* genomes (Table S1), we identified 287,010 parsimony-informative SNPs; the consistency index of the tree was 0.14, and the retention index was 0.38, suggesting substantial homoplasy, most likely due to recombination (44). The maximum likelihood phylogeny (Fig. S2), including the SNPs that went into its construction, was used to contextualize the placement of enrichments. Included in this genome set were isolates collected from lethal cases of Coccidioidomycosis in PTMs collected over a ~39-month time frame (Table 1). Genomes from WaNPRC PTMs grouped in multiple locations in the tree (Fig. S2), demonstrating the diversity of *C. posadasii* genotypes observed in a single location over time.

**TABLE 1:** Details of PTMs with fatal coccidioidomycosis

Sample	Source	First seropositivity
Z18179-RL	Macaque, right lung	12/9/20
Z18179-LL	Macaque, left lung	12/9/20
Z18179-S	Macaque, spleen	12/9/20
Z09114_RLL	Macaque, lower right lung	11/27/23
Z09114_RLU	Macaque, upper left lung	11/27/23
Z14352_LLM	Macaque, left lung middle	6/21/24
Z14352_T	Macaque, trachea	6/21/24
Z1523	Macaque	2/14/23
Z1640	Macaque	5/10/21

### Enrichment statistics

In this study, we successfully enriched and sequenced fourteen samples (extracts from two soil samples, nine air filters, and three animal tissue samples) (Table 2). Mapping raw sequence reads against Silveira returned a range of breadth values (≥5× depth), from ~0.1% to 56% (Table 2); 56% is ~16% more of the genome than was covered by the probes alone and can most likely be attributed to “by-catch” (45), or sequencing additional regions of the DNA that flank targeted sequence that are captured by probes. Our qPCR assay detected a marked increase in signal after enrichment, although the BOC across the reference genome, based on sequence data, did not always correlate. This could be due to preferential amplification, where our qPCR target was amplified and enriched more than the rest of the genome.

**TABLE 2:** Details of samples enriched in this study

Sample	Site	Sample	# Reads	Pre-Ct	Post-Ct	% Mapped to Silveira	% Breadth (5×)	Silveira genes covered (80%; 5×)^*a*^	Accession	Date collected
31 L^*c*^	Mesa	Rodent	13,991,878	34.61	18.93	50	43.12	5,380	SRR30946009	5/2022
14-2A^*c*^	Mesa	Soil	11,652,414	35.45	16.5	46	10.44	123	SRR30946008	7/2022
407-B16bi^*d*^	Tucson	Soil	38,856,026	35.36	19.95	97	45.72	6,235	SRR30946007	5/23/2018
CPA00032-Mouse^*b*^	Tucson	Rodent	86,316,628	24.5	20.71	92	50.85^*e*^	6,924	SRR30946005	N/A^*f*^
68 a^*b*^	Mesa	Filter	12,206,716	18	19.81	42	41.6	4,876	SRR33916357	11/2/23
68d^*b*^	Mesa	Filter	2,368,473	19.71	20.67	86	6.6	54	SRR33916356	11/2/23
66a^*c*^	Mesa	Filter	60,812,090	23.39	20.12	25	53.9	7,530	SRR33916355	10/21/23
69b^*c*^	Mesa	Filter	2,083,572	30.67	19.34	17	3.6	21	SRR33916354	11/8/23
66c^*c*^	Mesa	Filter	20,570,926	32.93	19.67	26	33.2	3,287	SRR33916353	10/21/23
69d^*d*^	Mesa	Filter	1,326,310	31.18	26.96	17	0.06	0	SRR33916352	11/8/23
69a^*d*^	Mesa	Filter	36,605,864	30.26	32.9	50	0.91	6	SRR34034339	11/8/23
68c^*d*^	Mesa	Filter	31,355,842	25.2	33.86	50	15.52	187	SRR34034338	11/2/23
67b^*d*^	Mesa	Filter	34,986,868	28.1	32.14	50	9.15	105	SRR34034337	10/27/23

^*a*^ 8,516 total CDSs.

^*b*^ 1 round of enrichment.

^*c*^ 2 rounds of enrichment.

^*d*^ 3 rounds of enrichment.

^*e*^ Spiked in controls.

^*f*^ N/A, not applicable.

### *WG-FAST* validation on paired WGS/enrichments data sets

To validate the *WG-FAST* approach on enrichment data, we placed both enriched reads (SRR30946005) and WGS reads (SRR30316899) from the same sample (*C. posadasii* CPA00032) into the global phylogeny. The results demonstrate that both the WGS reads and the enrichment reads placed CPA00032 near the reference genome assembly (Fig. S3). The CPA0032 genome has a longer branch length. As only parsimony-informative SNPs were called with *WG-FAST*, the SNPs leading to the CPA0032 genome are either autapomorphic or a product of recombination.

### *WG-FAST* performance on enrichments

**Fig 1:**
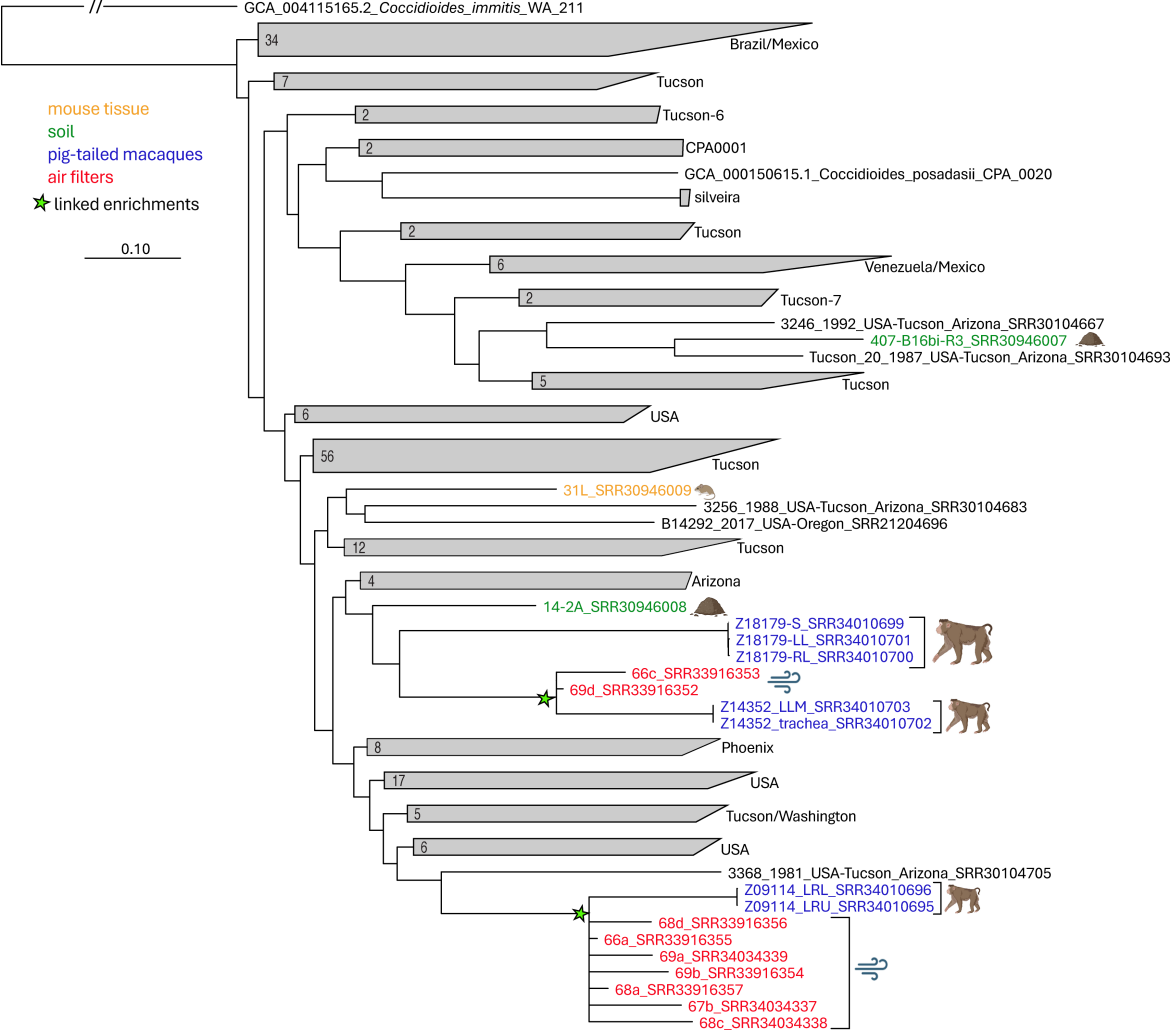
*WG-FAST* placement of enriched samples into the global population structure of *C. posadasii*. Clades that do not contain enrichments or PTM isolate genomes are collapsed and labeled by the consensus geographic location. Reads are also colored based on the isolation source of the enrichment. The green stars list nodes that include linked enrichments from different sources.

All enriched samples were placed by *WG-FAST* into the reference phylogeny (Fig. 1). A range of callable positions was observed (Table 3), resulting in variable levels of confidence in the resulting placement. Sample 407-B16bi-R3, enriched from Tucson soil, was placed with high confidence and grouped with other Tucson genomes. Two samples, one soil (14-2A) and one mouse tissue (31L), were both enriched from WaNPRC samples, although they were placed in different regions on the phylogeny. The Silveira enrichment from the mouse infection study was grouped with the known placement of Silveira, using a different reference genome for SNP discovery (Fig. S4).

**TABLE 3:** Details of samples inserted into the reference population with *WG-FAST*

Sample	# Callable sites^*a*^	# Polymorphic sites called^*b*^	Insertion likelihood
407-B16bi	172,502	13,179	100%
31L	94,372	5,784	100%
14-2A	8,989	575	99%
Sil-mouse^*c*^	165,116	1	85%
CPA00032-Mouse^*c*^	185,939	19,661	100%
68a	163,894	11,632	100%
69b	13,570	1,099	100%
68d	26,415	2,264	100%
66c	133,458	10,471	100%
69d	221	19	55%
66a	213,068	15,695	100%
69a	2,687	320	100%
68c	38,336	4,268	100%
67b	23,834	2,662	100%

^*a*^ Out of 287,010 sites in Silveira.

^*b*^ Minimum 2× depth, >80% proportion.

^*c*^ Only mapped reads.

Seven of the nine air filter enrichments grouped together on the phylogeny (Fig. 1), despite showing variable levels of enrichment success (Table 2). These seven enrichments shared a node with two isolates taken from the same PTM (Z09114); air filters were all collected in early November 2023, and the PTM became sick in late November 2023. All PTMs were housed within 17 m of the air sampling device.

The other two air filter enrichments (66c, 69d) were grouped with a different PTM (Z14352); this macaque became sick in June of 2024, whereas air filters were collected in late October and early November of 2023. Of the 1,086 SNPs that are unique to the branch that includes Z14352 isolates, only one of these SNPs was called in enrichment 69d, resulting in a less confident, but supported placement (Table 3) in the reference phylogeny. The enrichment data from a soil sample (14-2A) collected from the Mesa site in 2022 shared a most recent common ancestor with two different PTMs and these air filter enrichments (Fig. 1), suggesting a common, but differentiated origin for soil, air, and PTM samples.

### Gene content of a successful enrichment

The 407-B16bi-R3 soil enrichment from Tucson covered 73% of CDSs (*n* = 6,235) in *C. posadasii* str. Silveira, at an 80% BOC (5× minimum depth). When reads from 407-B16bi-R3 were aligned against Silveira with Snippy, 652 SNPs, 7 insertions, 4 deletions, and 9 “complex” mutations were identified. The genome assembly of mapped reads was 9.58 Mb (34% of Silveira assembly size) and was highly fragmented (>9,000 contigs), which would complicate *de novo*-based gene investigations.

### *In silico* limit of detection

To determine how few reads could be used for accurate genotyping, we mapped reads from HS-I-000233 (SRR14250212) to probe sequences, subsampled mapped reads, and placed them into the reference phylogeny with *WG-FAST*. The HS-I-000233 genome assembly was in the reference phylogeny, representing a phylogenetic anchor; previous work has demonstrated that phylogenetic anchors lower the number of required reads needed for accurate genotyping (38). The results of the current study demonstrate that as few as 200 paired-end (PE) simulated reads (250 nt) were able to accurately genotype a sample >97% of the time, based on 100 replicates (Table 4); as few as 300 PE reads were sufficient to genotype the sample 100% of the time. If this level of efficiency could be obtained through real sequence data, hundreds of enrichments could be sequenced on a single Illumina NextSeq run and still provide high-resolution genotyping.

**TABLE 4:** Limit of detection analysis based on sub-sampling and placing a known genotype of *C. posadasii*

Sample	# PE reads^*a*^	# Correctly placed subsets (*n* = 100)
HS-I-000233	100	84
HS-I-000233	200	97
HS-I-000233	300	100
HS-I-000233	400	100
HS-I-000233	500	100

^*a*^ 250 nucleotides.

## DISCUSSION

*C. posadasii* is an emerging fungal pathogen with an expanding but poorly understood geographic range. Culturing the fungus from the environment is very challenging, thus most isolates and genomes are biased to clinical cases. As such, the infectious potential of environmental strains is not fully understood. The unbiased analysis of DNA from environmental strains is expected to provide a holistic gene composition of *Coccidioides* spp., as well as identify disease transmission between environmental and mammalian reservoirs.

To provide researchers with additional tools for the characterization of *Coccidioides* from the environment, we designed and validated a probe enrichment system*.* The system was limited to CDSs from a single reference genome, based on a maximum number of RNA probes synthesized in a single design. The probe enrichment system is highly flexible, however, and new probes can be easily added to focus on specific regions of the genome, including regions outside of the annotated coding regions in Silveira. For example, the next planned iteration of this probe enrichment system will include probes specific to *C. immitis*, as well as those that target mating type genes, which are indicative of sexual recombination (46).

The probe enrichment system can provide information on gene composition in an enriched sample. For example, the enrichment of soil sample 407-B16bi-R3 from Tucson covered 73% of CDSs (*n* = 6,235) in Silveira, at an 80% BOC (5× minimum depth). Obtaining the CDS sequence allows for the in-depth investigation of SNPs, indels, and gene rearrangements that could affect the biology of the fungus; the fragmented nature of the enrichment assembly suggests that reference-guided assemblies will provide the most robust information on large structural rearrangements that could be biologically relevant.

The probe enrichment system also provides high-resolution genotyping directly from the sample. The enrichment of DNA from the deliberate infection of mouse tissues with two different reference strains of *C. posadasii* demonstrated that accurate, high-resolution genotyping could be obtained using partial SNP genotypes, as has been demonstrated for other pathogens (19, 38, 47). The probe enrichment system was then applied to DNA from one soil sample, one mouse tissue sample, and nine air filters, all collected from a site in Mesa, Arizona, that is consistently positive for the environmental detection of *C. posadasii*. The molecular detection of *Coccidioides* in the environment is frequently unsuccessful, even in endemic areas, such as Maricopa County, Arizona. Therefore, the Mesa site represents an important study site to understand the transmission, composition, and evolution of the pathogen over time. The site houses the WaNPRC, with PTMs caged outdoors that occasionally become naturally infected with Valley fever. Isolates collected from fatal PTM infections were sequenced to understand the diversity of strains circulating at the site over a ~39-month time frame. A phylogenetic analysis demonstrated that none of the PTM isolates from separate animals were identical (Fig. 1), suggesting that either the dominant *C. posadasii* genotype changes over time or that multiple genotypes are present endemically and occasionally become aerosolized; additional deep and consistent sampling at this site over time is needed to evaluate these hypotheses.

The low *Coccidioides* signal in the pre-enrichment samples from Mesa, based on published qPCR assays, was generally increased after one round of enrichment (Table 2). A soil enrichment (14-2A) and a mouse enrichment (31L) from the Mesa site returned different genotypes (Fig. 1). Seven of the air filter enrichments grouped with isolates obtained from PTM isolate Z09114. The air filters were collected ~3 weeks prior to the first reported case of PTM Coccidioidomycosis, which is approximately the incubation time of the disease (48). Two additional air filter enrichments (66C, 69d) grouped with an isolate obtained from a different case of lethal Coccidioidomycosis in a separate PTM (sample Z14352; Fig. 1), although the macaque became sick ~7 months after the air filter sample was collected; for enrichment 69d, only a single lineage-specific SNP was genotyped, which demonstrates the power of even single canonical SNPs for resolving strains (49). An additional three isolates from macaques with lethal Coccidioidomycosis were obtained from the Mesa site, although they were of different genotypes collected during different time windows (Table 1) and generally did not show close links with enrichments sequenced in this study (Fig. S3). Of note, our air sampling efforts captured relatively short time windows that may not have overlapped with the PTM infection. Regardless, this is the first case of demonstrating a clear epidemiological link between environmental detection and Coccidioidomycosis and provides a framework for future surveillance efforts.

*C. posadasii* sequencing efforts are heavily biased toward clinical strains. The ability to obtain high-resolution genotyping and gene content information from environmental strains, without the need to culture, is important to understand transmission dynamics, infection sources, and environmental spread. Enriching DNA from soil using RNA probes, such as those designed and employed in this study, is expensive and cannot be routinely performed in most cases and is not meant to be used as a screening or diagnostic tool. The detection of *Coccidioides* in the environment should be performed through rapid tests, such as qPCR. If genotyping is required, sub-genomic techniques, including targeted amplicon sequencing (47), could be used to obtain rough genotyping information and focus additional studies. Finally, probe enrichment could be used to not only obtain high-resolution genotyping information but also to answer important biological questions concerning gene content and genotype/phenotype associations. One limitation of all probe enrichment systems is when there are multiple genotypes from the same species present in a single sample. Mixed genotypes will all be enriched and sequenced and presented as mixed alleles across genotyped SNPs. Our previous testing demonstrates that these mixed samples can be identified through several metrics (19, 38), although untangling the mixtures through allele frequencies is not always possible. And although no mixtures were identified in the current study, the possibility of mixed genotypes, especially on air filters, is a likely possibility.

Obtaining sufficient enrichment data from environmental isolates will enable large comparative genomics studies (e.g., genome-wide association studies) to better understand differences between fungi from the environment and those from clinical cases, including those with differential virulence. As probe enrichment improves our understanding of environmental *Coccidioides* genomics, a better understanding of exposure risk will be obtained and guide mitigation efforts focused on reducing human exposure.

## Data Availability

All sequence data were submitted under BioProjects PRJNA1150245 (enrichments) and PRJNA1276095 (isolates from PTMs). Specific accession numbers are shown in Table S1.

## SUPPLEMENTAL MATERIAL

Supplemental Figures and Tables mbio.03396-25-s0001.pdf Fig. S1 to S4; Table S1 to S3.

ASM does not own the copyrights to Supplemental Material that may be linked to, or accessed through, an article. The authors have granted ASM a non-exclusive, world-wide license to publish the Supplemental Material files. Please contact the corresponding author directly for reuse.
