# Supplementary material for: Linking the epidemiology of coccidioidomycosis and environmental exposure through targeted genomic enrichment of Coccidioides posadasii
Source: mBio. 2025 Dec 30;17(2):e03396-25. doi: 10.1128/mbio.03396-25 (PMC12893014; doi:10.1128/mbio.03396-25)
Supplement: Supplemental Figures and Tables — Fig. S1 to S4; Table S1 to S3. [file mbio.03396-25-s0001.pdf]

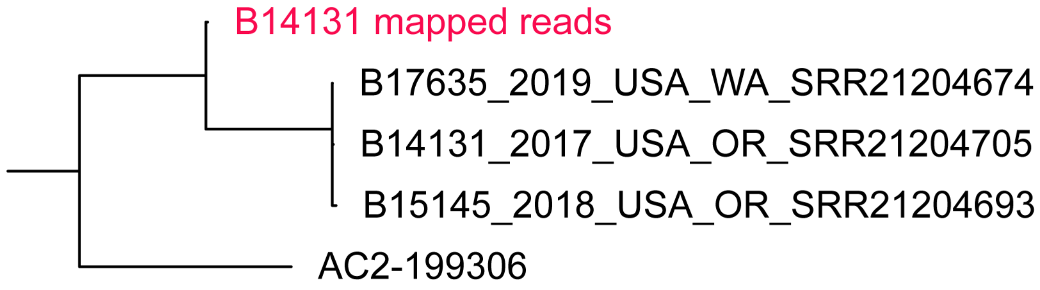

Figure S1. *WG-FAST* placement of *C. immitis* reads mapped against probes designed in this study. Reads from isolate B14131 (SRR21204705) were mapped against probes and only probes that mapped were exported and placed with *WG-FAST*. A subset of the larger *C. immitis* tree is shown.

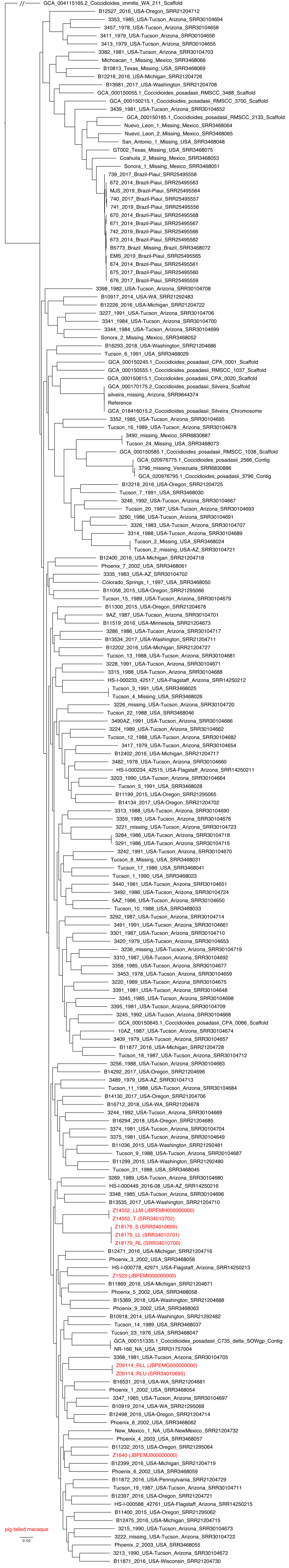

Figure S2. Phylogeny of the population structure of *C. posadasii* genomes used in this study. Single nucleotide polymorphisms (SNPs) were identified from genome assemblies with NASP and a maximum-likelihood phylogeny was inferred with IQ-TREE. Genomes generated in this study from pig-tailed macaque isolates are shown in red.

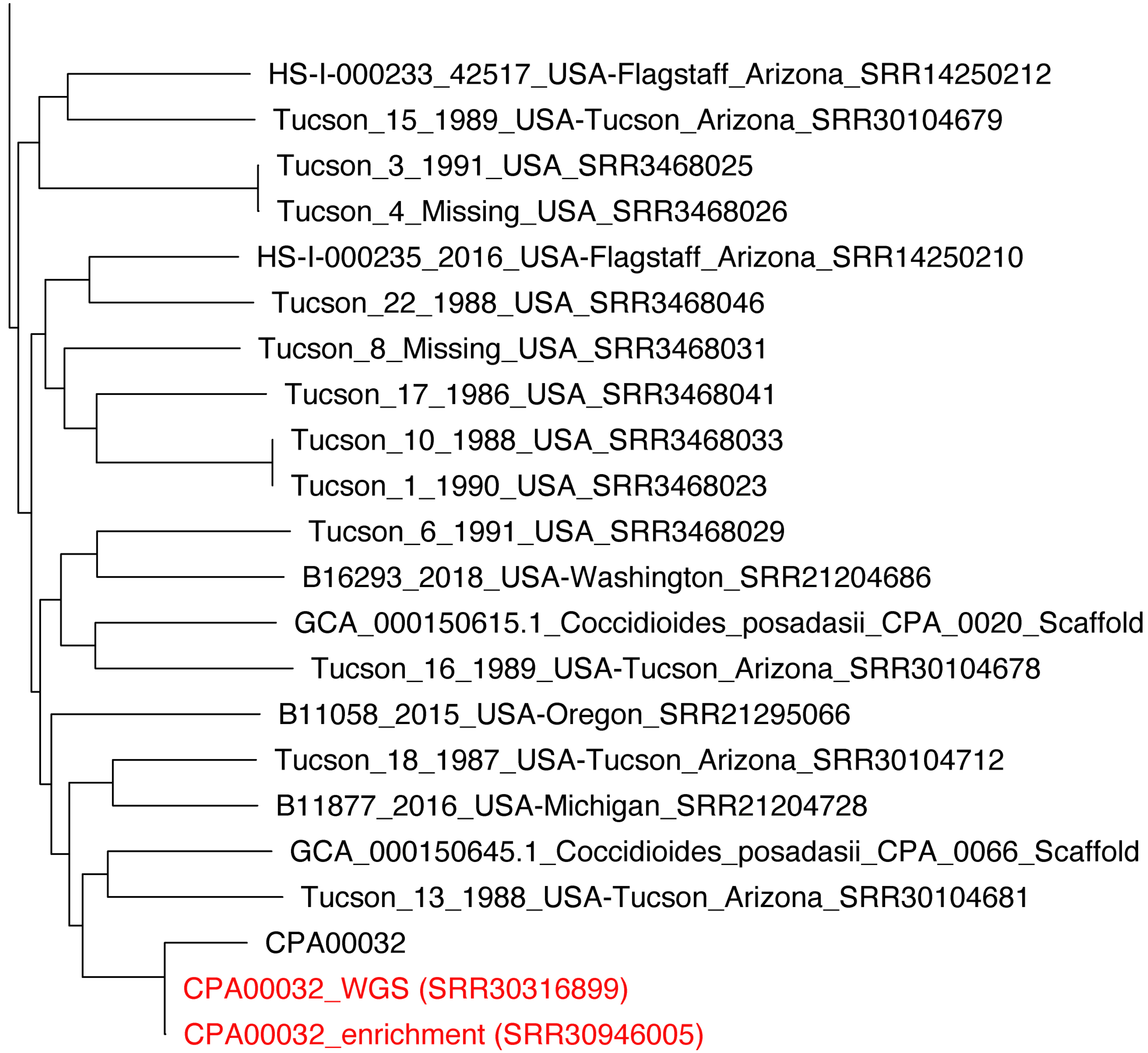

Figure S3. *WG-FAST* placement of enrichment reads and whole genome sequence reads of isolate CPA00032 into the reference phylogeny. CPA00032 came from a soil sample that was passaged through a mouse to obtain an isolate. The isolate was then used to infect a mouse, and the enrichment of that sample was sequenced and placed into the reference phylogeny with *WG-FAST*. The genome assembly for CPA00032 was part of the reference phylogeny and the whole genome sequence reads for CPA00032 were also placed into the reference tree with *WG-FAST*.

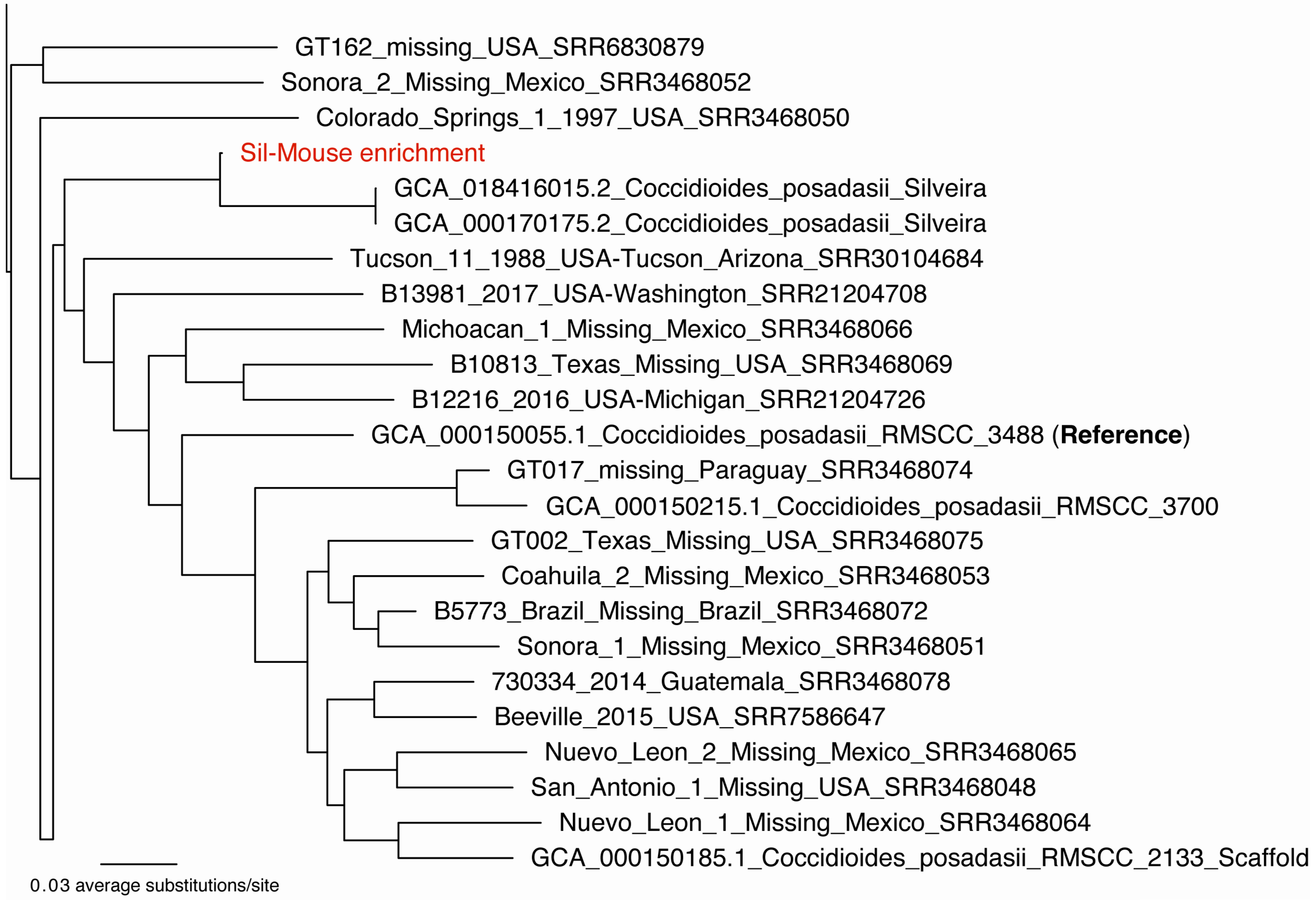

Figure S4. *WG-FAST* placement of enriched reads obtained from a laboratory mouse infection with *C. posadasii* str. Silveira. The reference genome used in this run was RMSCC-3488 (GCA_000150055.1).

Table S1: accession information for genomes analyzed in this study

Sample name	data type	Accession	Species
10AZ_1987_USA-Tucson_Arizona_SRR30104674	SRA	SRR30104674	posadasii
Z09114_Lung_R-Lower	SRA	SRR34010696	posadasii
Z09114_lung_R-upper	SRA	SRR34010695	posadasii
Z14352_lung_L-middle	SRA	SRR34010703	posadasii
Z14352_trachea	SRA	SRR34010702	posadasii
Z18179-LL	SRA	SRR34010701	posadasii
Z18179-RL	SRA	SRR34010700	posadasii
Z18179-S	SRA	SRR34010699	posadasii
3203_1990_USA-Tucson_Arizona_SRR30104664	SRA	SRR30104664	posadasii
3213_1990_USA-Tucson_Arizona_SRR30104672	SRA	SRR30104672	posadasii
3215_1990_USA-Tucson_Arizona_SRR30104673	SRA	SRR30104673	posadasii
3220_1989_USA-Tucson_Arizona_SRR30104675	SRA	SRR30104675	posadasii
3221_missing_USA-Tucson_Arizona_SRR30104723	SRA	SRR30104723	posadasii
3222_missing_USA-Tucson_Arizona_SRR30104722	SRA	SRR30104722	posadasii
3224_1989_USA-Tucson_Arizona_SRR30104662	SRA	SRR30104662	posadasii
3226_missing_USA-Tucson_Arizona_SRR30104720	SRA	SRR30104720	posadasii
3227_1991_USA-Tucson_Arizona_SRR30104706	SRA	SRR30104706	posadasii
3228_1991_USA-Tucson_Arizona_SRR30104671	SRA	SRR30104671	posadasii
3236_missing_USA-Tucson_Arizona_SRR30104719	SRA	SRR30104719	posadasii
3242_1991_USA-Tucson_Arizona_SRR30104670	SRA	SRR30104670	posadasii
3244_1992_USA-Tucson_Arizona_SRR30104669	SRA	SRR30104669	posadasii
3245_1992_USA-Tucson_Arizona_SRR30104668	SRA	SRR30104668	posadasii
3246_1992_USA-Tucson_Arizona_SRR30104667	SRA	SRR30104667	posadasii
3256_1988_USA-Tucson_Arizona_SRR30104683	SRA	SRR30104683	posadasii
3269_1989_USA-Tucson_Arizona_SRR30104680	SRA	SRR30104680	posadasii
3284_1986_USA-Tucson_Arizona_SRR30104718	SRA	SRR30104718	posadasii

3286_1986_USA-Tucson_Arizona_SRR30104717	SRA	SRR30104717	posadasii
3290_1986_USA-Tucson_Arizona_SRR30104691	SRA	SRR30104691	posadasii
3291_1986_USA-Tucson_Arizona_SRR30104715	SRA	SRR30104715	posadasii
3292_1987_USA-Tucson_Arizona_SRR30104714	SRA	SRR30104714	posadasii
3301_1987_USA-Tucson_Arizona_SRR30104710	SRA	SRR30104710	posadasii
3310_1987_USA-Tucson_Arizona_SRR30104692	SRA	SRR30104692	posadasii
3313_1988_USA-Tucson_Arizona_SRR30104690	SRA	SRR30104690	posadasii
3314_1988_USA-Tucson_Arizona_SRR30104689	SRA	SRR30104689	posadasii
3315_1988_USA-Tucson_Arizona_SRR30104688	SRA	SRR30104688	posadasii
3326_1983_USA-Tucson_Arizona_SRR30104707	SRA	SRR30104707	posadasii
3335_1983_USA-AZ_SRR30104702	SRA	SRR30104702	posadasii
3341_1984_USA-Tucson_Arizona_SRR30104700	SRA	SRR30104700	posadasii
3344_1984_USA-Tucson_Arizona_SRR30104699	SRA	SRR30104699	posadasii
3345_1985_USA-Tucson_Arizona_SRR30104698	SRA	SRR30104698	posadasii
3347_1985_USA-Tucson_Arizona_SRR30104697	SRA	SRR30104697	posadasii
3348_1985_USA-Tucson_Arizona_SRR30104696	SRA	SRR30104696	posadasii
3352_1985_USA-Tucson_Arizona_SRR30104695	SRA	SRR30104695	posadasii
3353_1985_USA-Tucson_Arizona_SRR30104694	SRA	SRR30104694	posadasii
3358_1985_USA-Tucson_Arizona_SRR30104677	SRA	SRR30104677	posadasii
3359_1985_USA-Tucson_Arizona_SRR30104676	SRA	SRR30104676	posadasii
3368_1981_USA-Tucson_Arizona_SRR30104705	SRA	SRR30104705	posadasii
3374_1981_USA-Tucson_Arizona_SRR30104704	SRA	SRR30104704	posadasii
3375_1981_USA-Tucson_Arizona_SRR30104649	SRA	SRR30104649	posadasii
3382_1981_USA-Tucson_Arizona_SRR30104703	SRA	SRR30104703	posadasii
3391_1981_USA-Tucson_Arizona_SRR30104648	SRA	SRR30104648	posadasii
3395_1981_USA-Tucson_Arizona_SRR30104709	SRA	SRR30104709	posadasii
3398_1982_USA-Tucson_Arizona_SRR30104708	SRA	SRR30104708	posadasii
3409_1979_USA-Tucson_Arizona_SRR30104657	SRA	SRR30104657	posadasii
3411_1979_USA-Tucson_Arizona_SRR30104656	SRA	SRR30104656	posadasii

3413_1979_USA-Tucson_Arizona_SRR30104655	SRA	SRR30104655	posadasii
3417_1979_USA-Tucson_Arizona_SRR30104654	SRA	SRR30104654	posadasii
3420_1979_USA-Tucson_Arizona_SRR30104653	SRA	SRR30104653	posadasii
3439_1981_USA-Tucson_Arizona_SRR30104652	SRA	SRR30104652	posadasii
3440_1981_USA-Tucson_Arizona_SRR30104651	SRA	SRR30104651	posadasii
3453_1978_USA-Tucson_Arizona_SRR30104659	SRA	SRR30104659	posadasii
3457_1978_USA-Tucson_Arizona_SRR30104658	SRA	SRR30104658	posadasii
3482_1978_USA-Tucson_Arizona_SRR30104660	SRA	SRR30104660	posadasii
3489_1979_USA-AZ_SRR30104713	SRA	SRR30104713	posadasii
3490AZ_1991_USA-Tucson_Arizona_SRR30104686	SRA	SRR30104686	posadasii
3490_missing_Mexico_SRR6830887	SRA	SRR6830887	posadasii
3491_1991_USA-Tucson_Arizona_SRR30104661	SRA	SRR30104661	posadasii
3492_1986_USA-Tucson_Arizona_SRR30104724	SRA	SRR30104724	posadasii
3796_missing_Venezuela_SRR6830886	SRA	SRR6830886	posadasii
5AZ_1986_USA-Tucson_Arizona_SRR30104650	SRA	SRR30104650	posadasii
670_2014_Brazil-Piaui_SRR25495568	SRA	SRR25495568	posadasii
671_2014_Brazil-Piaui_SRR25495567	SRA	SRR25495567	posadasii
672_2014_Brazil-Piaui_SRR25495563	SRA	SRR25495563	posadasii
673_2014_Brazil-Piaui_SRR25495562	SRA	SRR25495562	posadasii
674_2014_Brazil-Piaui_SRR25495561	SRA	SRR25495561	posadasii
675_2017_Brazil-Piaui_SRR25495560	SRA	SRR25495560	posadasii
676_2017_Brazil-Piaui_SRR25495559	SRA	SRR25495559	posadasii
739_2017_Brazil-Piaui_SRR25495558	SRA	SRR25495558	posadasii
740_2017_Brazil-Piaui_SRR25495557	SRA	SRR25495557	posadasii
741_2019_Brazil-Piaui_SRR25495556	SRA	SRR25495556	posadasii
742_2019_Brazil-Piaui_SRR25495566	SRA	SRR25495566	posadasii
9AZ_1987_USA-Tucson_Arizona_SRR30104701	SRA	SRR30104701	posadasii
B10813_Texas_Missing_USA_SRR3468069	SRA	SRR3468069	posadasii
B10917_2014_USA-WA_SRR21292483	SRA	SRR21292483	posadasii

B10918_2014_USA-Washington_SRR21292482	SRA	SRR21292482	posadasii
B10919_2014_USA-WA_SRR21295068	SRA	SRR21295068	posadasii
B11036_2015_USA-Washington_SRR21292481	SRA	SRR21292481	posadasii
B11058_2015_USA-Oregon_SRR21295066	SRA	SRR21295066	posadasii
B11199_2015_USA-Oregon_SRR21295065	SRA	SRR21295065	posadasii
B11232_2015_USA-Oregon_SRR21295064	SRA	SRR21295064	posadasii
B11299_2015_USA-Washington_SRR21292480	SRA	SRR21292480	posadasii
B11300_2015_USA-Oregon_SRR21204676	SRA	SRR21204676	posadasii
B11400_2015_USA-Oregon_SRR21295062	SRA	SRR21295062	posadasii
B11519_2016_USA-Minnesota_SRR21204673	SRA	SRR21204673	posadasii
B11869_2016_USA-Michigan_SRR21204671	SRA	SRR21204671	posadasii
B11871_2016_USA-Wisconsin_SRR21204730	SRA	SRR21204730	posadasii
B11872_2016_USA-Pennsylvania_SRR21204729	SRA	SRR21204729	posadasii
B11877_2016_USA-Michigan_SRR21204728	SRA	SRR21204728	posadasii
B12202_2016_USA-Michigan_SRR21204727	SRA	SRR21204727	posadasii
B12216_2016_USA-Michigan_SRR21204726	SRA	SRR21204726	posadasii
B12218_2016_USA-Oregon_SRR21204725	SRA	SRR21204725	posadasii
B12226_2016_USA-Michigan_SRR21204722	SRA	SRR21204722	posadasii
B12397_2016_USA-Oregon_SRR21204721	SRA	SRR21204721	posadasii
B12399_2016_USA-Michigan_SRR21204719	SRA	SRR21204719	posadasii
B12400_2016_USA-Michigan_SRR21204718	SRA	SRR21204718	posadasii
B12402_2016_USA-Michigan_SRR21204717	SRA	SRR21204717	posadasii
B12471_2016_USA-Michigan_SRR21204716	SRA	SRR21204716	posadasii
B12475_2016_USA-Michigan_SRR21204715	SRA	SRR21204715	posadasii
B12498_2016_USA-Oregon_SRR21204714	SRA	SRR21204714	posadasii
B12527_2016_USA-Oregon_SRR21204712	SRA	SRR21204712	posadasii
B13534_2017_USA-Washington_SRR21204711	SRA	SRR21204711	posadasii
B13535_2017_USA-Washington_SRR21204710	SRA	SRR21204710	posadasii
B13981_2017_USA-Washington_SRR21204708	SRA	SRR21204708	posadasii

B14130_2017_USA-Oregon_SRR21204706	SRA	SRR21204706	posadasii
B14134_2017_USA-Oregon_SRR21204702	SRA	SRR21204702	posadasii
B14292_2017_USA-Oregon_SRR21204696	SRA	SRR21204696	posadasii
B15369_2018_USA-Washington_SRR21204688	SRA	SRR21204688	posadasii
B16293_2018_USA-Washington_SRR21204686	SRA	SRR21204686	posadasii
B16294_2018_USA-Oregon_SRR21204685	SRA	SRR21204685	posadasii
B16531_2018_USA-WA_SRR21204681	SRA	SRR21204681	posadasii
B5773_Brazil_Missing_Brazil_SRR3468072	SRA	SRR3468072	posadasii
Coahuila_2_Missing_Mexico_SRR3468053	SRA	SRR3468053	posadasii
Colorado_Springs_1_1997_USA_SRR3468050	SRA	SRR3468050	posadasii
EMS_2019_Brazil-Piaui_SRR25495565	SRA	SRR25495565	posadasii
Coccidioides_posadasii_RMSCC_3488	Assembly	GCA_000150055.1	posadasii
Coccidioides_posadasii_RMSCC_2133_	Assembly	GCA_000150185.1	posadasii
Coccidioides_posadasii_RMSCC_3700_	Assembly	GCA_000150215.1	posadasii
Coccidioides_posadasii_CPA_0001_	Assembly	GCA_000150245.1	posadasii
Coccidioides_posadasii_RMSCC_1037_	Assembly	GCA_000150555.1	posadasii
Coccidioides_posadasii_RMSCC_1038	Assembly	GCA_000150585.1	posadasii
Coccidioides_posadasii_CPA_0020_	Assembly	GCA_000150615.1	posadasii
Coccidioides_posadasii_CPA_0066	Assembly	GCA_000150645.1	posadasii
Coccidioides_posadasii_C735_delta_SOWgp	Assembly	GCA_000151335.1	posadasii
Coccidioides_posadasii_Silveira	Assembly	GCA_000170175.2	posadasii
_Coccidioides_posadasii_Silveira	Assembly	GCA_018416015.2	posadasii
Coccidioides_posadasii_2566	Assembly	GCA_020976775.1	posadasii
Coccidioides_posadasii_3796	Assembly	GCA_020976795.1	posadasii
GT002_Texas_Missing_USA_SRR3468075	SRA	SRR3468075	posadasii
HS-I-000233_42517_USA-Flagstaff_Arizona_SRR14250212	SRA	SRR14250212	posadasii
HS-I-000234_42515_USA-Flagstaff_Arizona_SRR14250211	SRA	SRR14250211	posadasii
HS-I-000449_2016-08_USA-AZ_SRR14250216	SRA	SRR14250216	posadasii
HS-I-000588_42761_USA-Flagstaff_Arizona_SRR14250215	SRA	SRR14250215	posadasii

HS-I-000778_42971_USA-Flagstaff_Arizona_SRR14250213	SRA	SRR14250213	posadasii
MJS_2019_Brazil-Piaui_SRR25495564	SRA	SRR25495564	posadasii
Michoacan_1_Missing_Mexico_SRR3468066	SRA	SRR3468066	posadasii
NR-166_NA_USA_SRR31757004	SRA	SRR31757004	posadasii
New_Mexico_1_NA_USA-NewMexico_SRR21204732	SRA	SRR21204732	posadasii
Nuevo_Leon_1_Missing_Mexico_SRR3468064	SRA	SRR3468064	posadasii
Nuevo_Leon_2_Missing_Mexico_SRR3468065	SRA	SRR3468065	posadasii
Phoenix_1_2002_USA_SRR3468054	SRA	SRR3468054	posadasii
Phoenix_2_2003_USA_SRR3468055	SRA	SRR3468055	posadasii
Phoenix_3_2002_USA_SRR3468056	SRA	SRR3468056	posadasii
Phoenix_4_2003_USA_SRR3468057	SRA	SRR3468057	posadasii
Phoenix_5_2002_USA_SRR3468058	SRA	SRR3468058	posadasii
Phoenix_6_2002_USA_SRR3468059	SRA	SRR3468059	posadasii
Phoenix_7_2002_USA_SRR3468061	SRA	SRR3468061	posadasii
Phoenix_8_2002_USA_SRR3468062	SRA	SRR3468062	posadasii
Phoenix_9_2002_USA_SRR3468063	SRA	SRR3468063	posadasii
B16712_2018_USA_SRR21204678	SRA	SRR21204678	posadasii
San_Antonio_1_Missing_USA_SRR3468048	SRA	SRR3468048	posadasii
Sonora_1_Missing_Mexico_SRR3468051	SRA	SRR3468051	posadasii
Sonora_2_Missing_Mexico_SRR3468052	SRA	SRR3468052	posadasii
Tucson_10_1988_USA_SRR3468033	SRA	SRR3468033	posadasii
Tucson_11_1988_USA-Tucson_Arizona_SRR30104684	SRA	SRR30104684	posadasii
Tucson_12_1988_USA-Tucson_Arizona_SRR30104682	SRA	SRR30104682	posadasii
Tucson_13_1988_USA-Tucson_Arizona_SRR30104681	SRA	SRR30104681	posadasii
Tucson_14_1989_USA_SRR3468037	SRA	SRR3468037	posadasii
Tucson_15_1989_USA-Tucson_Arizona_SRR30104679	SRA	SRR30104679	posadasii
Tucson_16_1989_USA-Tucson_Arizona_SRR30104678	SRA	SRR30104678	posadasii
Tucson_17_1986_USA_SRR3468041	SRA	SRR3468041	posadasii
Tucson_18_1987_USA-Tucson_Arizona_SRR30104712	SRA	SRR30104712	posadasii

Tucson_19_1987_USA-Tucson_Arizona_SRR30104711	SRA	SRR30104711	posadasii
Tucson_1_1990_USA_SRR3468023	SRA	SRR3468023	posadasii
Tucson_20_1987_USA-Tucson_Arizona_SRR30104693	SRA	SRR30104693	posadasii
Tucson_21_1988_USA_SRR3468045	SRA	SRR3468045	posadasii
Tucson_22_1988_USA_SRR3468046	SRA	SRR3468046	posadasii
Tucson_23_1976_USA_SRR3468047	SRA	SRR3468047	posadasii
Tucson_24_Missing_USA_SRR3468073	SRA	SRR3468073	posadasii
Tucson_2_Missing_USA_SRR3468024	SRA	SRR3468024	posadasii
Tucson_2_missing_USA-AZ_SRR30104721	SRA	SRR30104721	posadasii
Tucson_3_1991_USA_SRR3468025	SRA	SRR3468025	posadasii
Tucson_4_Missing_USA_SRR3468026	SRA	SRR3468026	posadasii
Tucson_5_1991_USA_SRR3468028	SRA	SRR3468028	posadasii
Tucson_6_1991_USA_SRR3468029	SRA	SRR3468029	posadasii
Tucson_7_1991_USA_SRR3468030	SRA	SRR3468030	posadasii
Tucson_8_Missing_USA_SRR3468031	SRA	SRR3468031	posadasii
Tucson_9_1988_USA-Tucson_Arizona_SRR30104687	SRA	SRR30104687	posadasii
Z1523	SRA	SRR34010698	posadasii
Z1640	SRA	SRR34010697	posadasii
CPA00032	SRA	SRR30316899	posadasii
silveira_missing_Arizona_SRR9644374	SRA	SRR9644374	posadasii
Coccidioides_immitis_San_Joaquin_Valley_2	SRA	SRR1292218	*immitis*
Coccidioides_immitis_San_Joaquin_Valley_5	SRA	SRR1292219	*immitis*
Coccidioides_immitis_San_Joaquin_Valley_6	SRA	SRR1292220	*immitis*
Coccidioides_immitis_San_Joaquin_Valley_9	SRA	SRR1292221	*immitis*
Coccidioides_immitis_San_Joaquin_Valley_11	SRA	SRR1292222	*immitis*
Coccidioides_immitis_San_Diego_1	SRA	SRR1292223	*immitis*
Coccidioides_immitis_Washington_1	SRA	SRR1292224	*immitis*
Coccidioides_immitis_WA_202	SRA	SRR1292225	*immitis*

Coccidioides_immitis_WA_205	SRA	SRR1292226	*immitis*
Coccidioides_immitis_WA_211	SRA	SRR1292227	*immitis*
Coccidioides_immitis_WA_212	SRA	SRR1292228	*immitis*
Coccidioides_immitis_B17635	SRA	SRR21204674	*immitis*
Coccidioides_immitis_B17567	SRA	SRR21204675	*immitis*
Coccidioides_immitis_B17554	SRA	SRR21204677	*immitis*
Coccidioides_immitis_B16692	SRA	SRR21204679	*immitis*
Coccidioides_immitis_B16536	SRA	SRR21204680	*immitis*
Coccidioides_immitis_B16534	SRA	SRR21204682	*immitis*
Coccidioides_immitis_B16339	SRA	SRR21204683	*immitis*
Coccidioides_immitis_B16338	SRA	SRR21204684	*immitis*
Coccidioides_immitis_B11057	SRA	SRR21204687	*immitis*
Coccidioides_immitis_B15368	SRA	SRR21204689	*immitis*
Coccidioides_immitis_B15317	SRA	SRR21204690	*immitis*
Coccidioides_immitis_B15257	SRA	SRR21204691	*immitis*
Coccidioides_immitis_B15146	SRA	SRR21204692	*immitis*
Coccidioides_immitis_B15145	SRA	SRR21204693	*immitis*
Coccidioides_immitis_B15142	SRA	SRR21204694	*immitis*
Coccidioides_immitis_B14298	SRA	SRR21204695	*immitis*
Coccidioides_immitis_A502-2	SRA	SRR21204698	*immitis*
Coccidioides_immitis_B14288	SRA	SRR21204699	*immitis*
Coccidioides_immitis_B14286	SRA	SRR21204700	*immitis*
Coccidioides_immitis_B14135	SRA	SRR21204701	*immitis*
Coccidioides_immitis_B14133	SRA	SRR21204703	*immitis*
Coccidioides_immitis_B14132	SRA	SRR21204704	*immitis*
Coccidioides_immitis_B14131	SRA	SRR21204705	*immitis*
Coccidioides_immitis_A502-1	SRA	SRR21204709	*immitis*
Coccidioides_immitis_B12526	SRA	SRR21204713	*immitis*
Coccidioides_immitis_A432	SRA	SRR21204720	*immitis*

Coccidioides_immitis_B12220	SRA	SRR21204723	*immitis*
Coccidioides_immitis_B12219	SRA	SRR21204724	*immitis*
Coccidioides_immitis_A391	SRA	SRR21204731	*immitis*
Coccidioides_immitis_WA221	SRA	SRR21292664	*immitis*
Coccidioides_immitis_B11343	SRA	SRR21295063	*immitis*
Coccidioides_immitis_B11002	SRA	SRR21295067	*immitis*
Coccidioides_immitis_AC2-5501	SRA	SRR24010540	*immitis*
Coccidioides_immitis_AC2-199306	SRA	SRR24010552	*immitis*
Coccidioides_immitis_294	SRA	SRR25621432	*immitis*
Coccidioides_immitis_293	SRA	SRR25626827	*immitis*
Coccidioides_immitis_295	SRA	SRR25626839	*immitis*
Coccidioides_immitis_556	SRA	SRR25635497	*immitis*
Coccidioides_immitis_SJV1	SRA	SRR3468015	*immitis*
Coccidioides_immitis_SJV2	SRA	SRR3468016	*immitis*
Coccidioides_immitis_SJV11	SRA	SRR3468017	*immitis*
Coccidioides_immitis_Guerrero1	SRA	SRR3468018	*immitis*
Coccidioides_immitis_SanDiego1	SRA	SRR3468019	*immitis*
Coccidioides_immitis_Michoacan2	SRA	SRR3468020	*immitis*
Coccidioides_immitis_Coahuila1	SRA	SRR3468021	*immitis*
Coccidioides_immitis_B0727_Argentina	SRA	SRR3468022	*immitis*
Coccidioides_immitis_SJV3	SRA	SRR3468027	*immitis*
Coccidioides_immitis_SJV4	SRA	SRR3468038	*immitis*
Coccidioides_immitis_SJV5	SRA	SRR3468049	*immitis*
Coccidioides_immitis_SJV6	SRA	SRR3468060	*immitis*
Coccidioides_immitis_SJV7	SRA	SRR3468071	*immitis*
Coccidioides_immitis_SJV8	SRA	SRR3468079	*immitis*
Coccidioides_immitis_SJV9	SRA	SRR3468080	*immitis*
Coccidioides_immitis_SJV10	SRA	SRR3468081	*immitis*
Coccidioides_immitis_B12496	SRA	SRR7206598	*immitis*

Coccidioides_immitis_B13956	SRA	SRR7206599	*immitis*
Coccidioides_immitis_B12398	SRA	SRR7206600	*immitis*
Coccidioides_immitis_B12495	SRA	SRR7206601	*immitis*
Coccidioides_immitis_B11873	SRA	SRR7206603	*immitis*
Coccidioides_immitis_B11518	SRA	SRR7206604	*immitis*
Coccidioides_immitis_B11587	SRA	SRR7206605	*immitis*
Coccidioides_immitis_B11080	SRA	SRR7206606	*immitis*
Coccidioides_immitis_B11035	SRA	SRR8530933	*immitis*
Coccidioides_immitis_B11198	SRA	SRR8530934	*immitis*
Coccidioides_immitis_B11517	SRA	SRR8530935	*immitis*
Coccidioides_immitis_B11019	SRA	SRR8530936	*immitis*
Coccidioides_immitis_B11034	SRA	SRR8530937	*immitis*
Coccidioides_immitis_RS	Assembly	GCA_000149335.2	*immitis*
Coccidioides_immitis_H538_4	Assembly	GCA_000149815.1	*immitis*
Coccidioides_immitis_RMSCC_2394	Assembly	GCA_000149895.1	*immitis*
Coccidioides_immitis_RMSCC_3703	Assembly	GCA_000150085.1	*immitis*
Coccidioides_immitis_WA_211	Assembly	GCA_004115165.2	*immitis*

Table S2: Regions of the Silveira genome that contains probes that are unique to *Coccidioides posadasii*

lcl|CP075068.1_cds_QVM05400.1_110_[locus_tag=D8B26_000111]_[protein=hypothetical_protein]_[protein_id=QVM05400.1]
lcl|CP075068.1_cds_QVM05401.1_111_[locus_tag=D8B26_000112]_[protein=hypothetical_protein]_[protein_id=QVM05401.1]
lcl|CP075068.1_cds_QVM05461.1_171_[locus_tag=D8B26_000170]_[protein=hypothetical_protein]_[protein_id=QVM05461.1]
lcl|CP075068.1_cds_QVM05650.1_360_[locus_tag=D8B26_000357]_[protein=hypothetical_protein]_[protein_id=QVM05650.1]
lcl|CP075068.1_cds_QVM06269.1_979_[locus_tag=D8B26_000981]_[protein=hypothetical_protein]_[protein_id=QVM06269.1]
lcl|CP075068.1_cds_QVM06335.1_1045_[locus_tag=D8B26_001047]_[protein=hypothetical_protein]_[protein_id=QVM06335.1]
lcl|CP075068.1_cds_QVM06336.1_1046_[locus_tag=D8B26_001048]_[protein=hypothetical_protein]_[protein_id=QVM06336.1]
lcl|CP075068.1_cds_QVM06438.1_1148_[gene=SUB2_1]_[locus_tag=D8B26_001149]_[protein_id=QVM06438.1]
lcl|CP075068.1_cds_QVM06688.1_1398_[locus_tag=D8B26_001395]_[protein=hypothetical_protein]_[protein_id=QVM06688.1]
lcl|CP075068.1_cds_QVM06863.1_1573_[locus_tag=D8B26_001567]_[protein=hypothetical_protein]_[protein_id=QVM06863.1]
lcl|CP075068.1_cds_QVM07005.1_1715_[locus_tag=D8B26_001707]_[protein=hypothetical_protein]_[protein_id=QVM07005.1]
lcl|CP075068.1_cds_QVM07612.1_2322_[locus_tag=D8B26_002310]_[protein=hypothetical_protein]_[protein_id=QVM07612.1]
lcl|CP075068.1_cds_QVM07613.1_2323_[locus_tag=D8B26_002311]_[protein=hypothetical_protein]_[protein_id=QVM07613.1]
lcl|CP075068.1_cds_QVM07705.1_2415_[locus_tag=D8B26_002397]_[protein=hypothetical_protein]_[protein_id=QVM07705.1]
lcl|CP075068.1_cds_QVM07784.1_2494_[locus_tag=D8B26_002475]_[protein=hypothetical_protein]_[protein_id=QVM07784.1]
lcl|CP075068.1_cds_QVM07786.1_2496_[locus_tag=D8B26_002477]_[protein=hypothetical_protein]_[protein_id=QVM07786.1]
lcl|CP075068.1_cds_QVM07788.1_2498_[locus_tag=D8B26_002479]_[protein=hypothetical_protein]_[protein_id=QVM07788.1]
lcl|CP075068.1_cds_QVM07789.1_2499_[locus_tag=D8B26_002480]_[protein=hypothetical_protein]_[protein_id=QVM07789.1]
lcl|CP075069.1_cds_QVM08174.1_2884_[locus_tag=D8B26_002868]_[protein=hypothetical_protein]_[protein_id=QVM08174.1]
lcl|CP075069.1_cds_QVM08461.1_3171_[locus_tag=D8B26_003151]_[protein=hypothetical_protein]_[protein_id=QVM08461.1]
lcl|CP075069.1_cds_QVM08462.1_3172_[locus_tag=D8B26_003152]_[protein=hypothetical_protein]_[protein_id=QVM08462.1]
lcl|CP075069.1_cds_QVM08521.1_3231_[locus_tag=D8B26_003210]_[protein=hypothetical_protein]_[protein_id=QVM08521.1]
lcl|CP075069.1_cds_QVM08553.1_3263_[locus_tag=D8B26_003239]_[protein=hypothetical_protein]_[protein_id=QVM08553.1]
lcl|CP075069.1_cds_QVM08554.1_3264_[locus_tag=D8B26_003240]_[protein=hypothetical_protein]_[protein_id=QVM08554.1]
lcl|CP075069.1_cds_QVM08681.1_3391_[locus_tag=D8B26_003361]_[protein=hypothetical_protein]_[protein_id=QVM08681.1]
lcl|CP075069.1_cds_QVM08892.1_3602_[locus_tag=D8B26_003564]_[protein=hypothetical_protein]_[protein_id=QVM08892.1]
lcl|CP075069.1_cds_QVM08894.1_3604_[locus_tag=D8B26_003565]_[protein=hypothetical_protein]_[protein_id=QVM08894.1]
lcl|CP075069.1_cds_QVM09185.1_3895_[locus_tag=D8B26_003850]_[protein=hypothetical_protein]_[protein_id=QVM09185.1]
lcl|CP075069.1_cds_QVM09191.1_3901_[locus_tag=D8B26_003855]_[protein=hypothetical_protein]_[protein_id=QVM09191.1]
lcl|CP075069.1_cds_QVM09325.1_4035_[locus_tag=D8B26_003988]_[protein=hypothetical_protein]_[protein_id=QVM09325.1]
lcl|CP075069.1_cds_QVM09326.1_4036_[locus_tag=D8B26_003989]_[protein=hypothetical_protein]_[protein_id=QVM09326.1]
lcl|CP075069.1_cds_QVM09327.1_4037_[locus_tag=D8B26_003990]_[protein=hypothetical_protein]_[protein_id=QVM09327.1]
lcl|CP075069.1_cds_QVM09498.1_4208_[locus_tag=D8B26_004158]_[protein=hypothetical_protein]_[protein_id=QVM09498.1]
lcl|CP075069.1_cds_QVM09501.1_4211_[locus_tag=D8B26_004161]_[protein=hypothetical_protein]_[protein_id=QVM09501.1]
lcl|CP075069.1_cds_QVM09757.1_4467_[locus_tag=D8B26_004416]_[protein=hypothetical_protein]_[protein_id=QVM09757.1]
lcl|CP075069.1_cds_QVM09758.1_4468_[locus_tag=D8B26_004417]_[protein=hypothetical_protein]_[protein_id=QVM09758.1]
lcl|CP075069.1_cds_QVM09876.1_4586_[locus_tag=D8B26_004536]_[protein=hypothetical_protein]_[protein_id=QVM09876.1]
lcl|CP075069.1_cds_QVM10005.1_4715_[locus_tag=D8B26_004668]_[protein=hypothetical_protein]_[protein_id=QVM10005.1]
lcl|CP075069.1_cds_QVM10006.1_4716_[locus_tag=D8B26_004669]_[protein=hypothetical_protein]_[protein_id=QVM10006.1]
lcl|CP075069.1_cds_QVM10010.1_4720_[locus_tag=D8B26_004673]_[protein=hypothetical_protein]_[protein_id=QVM10010.1]
lcl|CP075069.1_cds_QVM10202.1_4912_[locus_tag=D8B26_004863]_[protein=hypothetical_protein]_[protein_id=QVM10202.1]
lcl|CP075070.1_cds_QVM10342.1_5052_[locus_tag=D8B26_005002]_[protein=hypothetical_protein]_[protein_id=QVM10342.1]
lcl|CP075070.1_cds_QVM10794.1_5504_[locus_tag=D8B26_005447]_[protein=hypothetical_protein]_[protein_id=QVM10794.1]
lcl|CP075070.1_cds_QVM10899.1_5609_[locus_tag=D8B26_005550]_protein=hypothetical_protein]_[protein_id=QVM10899.1]
lcl|CP075070.1_cds_QVM11185.1_5895_[locus_tag=D8B26_005835]_[protein=hypothetical_protein]_[protein_id=QVM11185.1]
lcl|CP075070.1_cds_QVM11555.1_6265_[locus_tag=D8B26_006202]_[protein=hypothetical_protein]_[protein_id=QVM11555.1]
lcl|CP075070.1_cds_QVM12190.1_6900_[locus_tag=D8B26_006825]_[protein=hypothetical_protein]_[protein_id=QVM12190.1]
lcl|CP075070.1_cds_QVM12257.1_6967_[locus_tag=D8B26_006890]_[protein=hypothetical_protein]_[protein_id=QVM12257.1]
lcl|CP075070.1_cds_QVM12259.1_6969_[locus_tag=D8B26_006892]_[protein=hypothetical_protein]_[protein_id=QVM12259.1]
lcl|CP075070.1_cds_QVM12353.1_7063_[locus_tag=D8B26_006983]_[protein=hypothetical_protein]_[protein_id=QVM12353.1]
lcl|CP075070.1_cds_QVM12354.1_7064_[locus_tag=D8B26_006984]_[protein=hypothetical_protein]_[protein_id=QVM12354.1]
lcl|CP075070.1_cds_QVM12356.1_7066_[locus_tag=D8B26_006986]_[protein=hypothetical_protein]_[protein_id=QVM12356.1]
lcl|CP075070.1_cds_QVM12357.1_7067_[locus_tag=D8B26_006987]_[protein=hypothetical_protein]_[protein_id=QVM12357.1]
lcl|CP075071.1_cds_QVM12487.1_7197_[locus_tag=D8B26_007112]_protein=hypothetical_protein]_[protein_id=QVM12487.1]
lcl|CP075071.1_cds_QVM12502.1_7212_[locus_tag=D8B26_007126]_[protein=hypothetical_protein]_[protein_id=QVM12502.1]
lcl|CP075071.1_cds_QVM12670.1_7380_[locus_tag=D8B26_007288]_[protein=hypothetical_protein]_[protein_id=QVM12670.1]
lcl|CP075071.1_cds_QVM12827.1_7537_[locus_tag=D8B26_007444]_[protein=hypothetical_protein]_[protein_id=QVM12827.1]
lcl|CP075071.1_cds_QVM12841.1_7551_[locus_tag=D8B26_007459]_[protein=hypothetical_protein]_[protein_id=QVM12841.1]
lcl|CP075072.1_cds_QVM13458.1_8168_[locus_tag=D8B26_008066]_[protein=hypothetical_protein]_[protein_id=QVM13458.1]
lcl|CP075072.1_cds_QVM13459.1_8169_[locus_tag=D8B26_008067]_[protein=hypothetical_protein]_[protein_id=QVM13459.1]
lcl|CP075072.1_cds_QVM13460.1_8170_[locus_tag=D8B26_008068]_[protein=hypothetical_protein]_[protein_id=QVM13460.1]

Table S3: The number of *C. posadasii* specific probes that were covered by enriched datasets.

Sample	*C. posadasii* specific probes covered*
407_B16bi_R3	422
14-2A	11
31L	110
66a	470
66c	316
67b	97
68a	245
68c	167
69d	0
69a	0
69b	39
68d	18

*≥80 breadth at ≥3x depth
